# Transforming health care: the policy and politics of service reconfiguration in the UK's four health systems

**DOI:** 10.1017/S1744133119000148

**Published:** 2020-07

**Authors:** Ellen Stewart, Scott L. Greer, Angelo Ercia, Peter D. Donnelly

**Affiliations:** 1 Centre for Biomedicine, Self & Society, Usher Institute, Old Medical School, University of Edinburgh, Teviot Place, Edinburgh EH8 9AG, UK; 2 School of Public Health, University of Michigan, 1415 Washington Heights, Ann Arbor, Michigan 48109-2029, USA; 3 Centre for Health Informatics, The University of Manchester, Vaughan House, Portsmouth Street, Manchester M13 9GB, UK; 4 Institute of Health Policy, Management and Evaluation, Dalla Lana School of Public Health, University of Toronto, Toronto, Canada

**Keywords:** Public involvement, change management, devolution, UK, health policy

## Abstract

Public involvement in service change has been identified as a key facilitator of health care transformation (Foley *et al*., 2017) but little is known about how health policy influences whether and how organisations involve the public in change processes. This qualitative study compares policy and practice for involving the public in major service changes across the UK's four health systems (England, Northern Ireland, Wales and Scotland). We analysed policy documents, and conducted interviews with officials, stakeholders, NHS staff and public campaigners (total number of interviewees = 47). Involving the public in major service change was acknowledged as a policy challenge in all four systems. Despite ostensible similarities, there were some clear differences between the four health systems' processes for involving patients and the public in major changes to health services. The extent of central Government oversight, the prescriptiveness of Government guidance, the role for intermediary bodies and arrangements for independent scrutiny of contentious decisions all vary. We analyse how health policy in the four systems has used ‘sticks’ and ‘sermons’ to promote particular approaches, and conclude that both policy and the wider system context within which health care organisations try to effect change are significant, and understudied aspect of contemporary practice.

The need for substantial changes to how we deliver health care – shifting budgets to prevention, accommodating new technologies and treating people in their own homes – is a near-orthodoxy among health policy elites in high-income health systems (Committee on the Quality of Health Care in America, 2001; Ham, 2006; Crisp *et al*., 2016). This orthodoxy is less widely shared, however, among the populations of high-income countries. Efforts to change the infrastructure of care, particularly any efforts that involve the closure of services, are frequently contentious. Perhaps as a result, the UK, with on paper some of the most centralised health systems to be found, has – since the widespread closure of long-stay institutions in the 1970s and 1980s (McCrone and Becker, 2000) – seen its various health reforms produce relatively modest changes in the local bricks and mortar of acute care delivery (Imison, 2011). It might be relatively easy to reorganise NHS management, but closing a hospital does not seem to be easy at all.

Real or anticipated public opposition to major changes to hospital services has been reported internationally (Goyder, 1999; Abelson, 2001; Fougere, 2001; Lepnurm and Lepnurm, 2001; Barnett and Barnett, 2003; Lindbom, 2014). In the UK, this has been described in academic studies as far back as the 1960s (Jones, 2015; Pettigrew *et al*., 1988) and continues to loom large (Fulop *et al*., 2012; Jones and Exworthy, 2015; Fraser *et al*., 2017; Daniels *et al*., 2018). A handful of recent academic publications concentrate on the specific challenge of understanding public perspectives on and improving public involvement within major service change in the UK (Barratt *et al*., 2015; Dalton *et al*., 2016; Foley *et al*., 2017). These have emphasised that “local communities and commissioners appear to operate in different paradigms of understanding about risk, when a reconfiguration is being discussed” (Barratt *et al*., 2015) and that “problems often arise because decision-makers paid insufficient attention to issues considered important by patients and the public” (Dalton *et al*., 2016). This might be seen as a part of the general phenomenon identified by Pierson (1996) in which welfare state investments produce their own constituencies; in this case, the staff, communities and patients who defend a hospital.

Responding to calls to acknowledge the political context of both change processes (Best *et al*., 2012), and public involvement processes (Madden and Speed, 2017), this paper explores public involvement in service change as a policy challenge, by comparing public involvement and service reconfiguration policies in the UK's four health systems (England, Wales, Northern Ireland and Scotland). Our specific, comparative, interest here is in policy: *how has health policy in each system sought to influence organisational approaches to involving the public in change*? Our research project used qualitative methods to study both policies in the four UK health systems, and specific reconfiguration projects around the UK in a purposive ‘most different systems’ design that maximised variation among cases (Stake, 2013).

Across all four systems, we found examples where change proposals led to political conflict and were never implemented, often damaging relationships along the way. But we also found significant variation in the extent to which the UK's four health systems were designed to promote involvement, encourage change or insulate the elected government from local reconfiguration conflicts. While much of the literature on change management and public involvement focuses on local actors such as managers, and local strategies such as consultations, we found that local NHS organisations plan and enact public involvement in response to, and in some systems under the close scrutiny of, national-level policy actors.

## 1. Policy instruments for public involvement in major service change

The literature on public involvement in health services is dominated by case studies of how particular organisations have planned and conducted activities, and, as others have noted, this can contribute to a tendency within the field to present public involvement as a technical exercise, rather than as a policy area with multiple, sometimes competing imperatives (Madden and Speed, 2017; Stewart, 2013b). Powell (2016, p. 113) states that “while many studies point to the importance of ‘public involvement’ in health care, most reviews are at the micro and meso levels, with little material on the macro/strategic level”. A recent special issue comparing public involvement in health care priority-setting cross-nationally pointed towards the relevance of institutionalised opportunity structures for public involvement, not in dictating whether or not the public would have a say in decisions, but in channelling their views through decision-making processes differently (Hunter *et al*., 2016). The sub-field of studies of public involvement in major health care change has also paid minimal attention to policy questions, focusing thus far on various local actors' perceptions of change (Fulop *et al*., 2012; Barratt *et al*., 2015; Foley *et al*., 2017). Acknowledging the presence of a, usually statutory, requirement for public authorities to involve the public in major service changes tends to be the limit of academic analyses of health policy in this area. We seek to redress this omission by focusing on differences between the macro/strategic level of legislation and policy guidance from the top of the political system. Acknowledging that policy creates a set of opportunity structures with wider consequences, we compare the four UK health systems in order to better understand what kinds of policy tools and approaches have been used, and with what effects.

The study of policy tools is a broad field in itself, and in this paper, we use one classification thereof to provide the first analysis comparing policy for public involvement in service change across the devolved health systems of the UK. Recognising the complexity intrinsic in this study, we chose one of the most parsimonious typologies of policy instruments which classifies tools as carrots (incentives), sticks (regulation) and sermons (information) (Bemelmans-Videc *et al*., 2003). The extent to which rhetoric across all four systems about the importance of public involvement in major change was accompanied by any policy tools to accomplish it is telling in itself. Beyond that, the relative packaging and choice of particular approaches allow us to explore the priority given to public involvement, and in the absence of agreed upon goals for this area of policy, to organise and compare the effects of these tools within their respective health systems. This recognises that choice of tools tells us little, absent a full analysis of what happens when policy encounters a health system's ‘large and often largely autonomous collection of actors’ (Peters, 2000).

The next section explains our study setting, comparative design and methods. The following sections then outline the four systems' different approaches to public involvement in service change, and describe the system-level policy contexts, on paper and in the words of interviewees. The discussion focuses on how policy in each of the four systems employs ‘sticks’ and ‘sermons’ (Bemelmans-Videc *et al*., 2003) to interact with local contexts, shaping understandings of both the means and ends of involvement.

## 2. Methods and settings

### 2.1 Settings

Our study context is the four different UK health systems of England, Northern Ireland, Scotland and Wales. They have had a high degree of political autonomy since 1998 and have diverged in significant respects (Greer, 2004, 2016; Birrell, 2012; Cylus *et al*., 2015; OECD, 2016). There are several asymmetries to start with. The population of England is around 53 million, while Northern Ireland (1.8 million), Wales (3 million) and Scotland (5.3 million) are far smaller. There is no significant regional level in the English NHS, making it by far the largest single health system of the western countries. The English NHS is also directly run by the UK government, which is chosen by voters of the whole UK. The three devolved systems are run by their own elected governments, each of which has a budgetary window set by the UK government with some tax autonomy for Scotland. The devolved governments are principally concerned with education, local government, criminal justice and health while the UK government additionally deals with issues such as foreign policy. This means that a local hospital conflict that would never make the English political agenda, especially if outside London, can easily become a national political debate in the devolved systems. Finally, devolved and English party systems are very distinct, with Scotland in the hands of the Scottish National Party for the last decade, Wales run by Labour, sometimes in coalition, for its whole devolved history, and Northern Ireland governed by its own parties in a complex power-sharing arrangement that has been suspended from January 2017 as of the time of writing, leaving it governed directly by the UK government.

At the time of our study in 2016, the three systems of the devolved governments, Northern Ireland, Scotland and Wales, had broadly converged on a simple and flat organisational structure (Greer, 2016). Below the central ministerial department, the bulk of each system is built around territorial units (boards in Scotland and Wales, trusts in Northern Ireland) that are responsible for most health provision in their areas. In Scotland and Wales, there is a direct chain of management from the minister to the department to the boards. In Northern Ireland, a fiction of ‘commissioning’ is preserved in which a single commissioning body responsible to the department shops for services from a set of integrated territorial trusts akin to the boards, but the system is fundamentally non-competitive. In all three cases, the NHS managers who decide the shape of services are clearly and practically accountable to ministers who are accountable to voters. Public involvement structures vary in their specifics (Tritter, 2011) but it is often assumed that the devolved systems have greater potential for public involvement given that citizens can seek to influence a single organisation which has a coherent overview of services across a locale (Ham *et al*., 2013; Stewart, 2013a) and that citizens have easier direct access to central politicians.

England by contrast has a complex system that reflects decades of work by governments of both parties to institute market mechanisms (Exworthy and Mannion, 2016; Bevir and Waring, 2017). The building blocks of the current system are ‘trusts’, which sell services at fixed prices to ‘commissioners’ who are either groups of local doctors, called Clinical Commissioning Groups, or the large national commissioner NHS England. A quality regulator and a financial regulator exist to ensure competition and financial balance. In response to austerity, NHS England began to orchestrate ‘Sustainability and Transformation Plans’ (STPs), in which local commissioners, trusts and NHS England agree on ways to integrate care and shift care out of hospitals with the hope of saving money and preserving quality (Walshe, 2017). The private sector continues to compete for services. Accountability for decisions is unclear and spread across NHS England, local commissioners and trusts. This fragmented structure, combined with the repeated reforms of local mechanisms of involvement, is widely perceived as an impeding effective public involvement (Martin and Carter, 2017).

### 2.2 Methods

Our study combines documentary analysis, semi-structured interviews with key policymakers and case studies of particular reconfigurations in each system. Our focus is on policy, and the majority of our data come from interviews and document analysis at the national level. We included a selection of illustrative case studies of particular reconfigurations to illustrate how a policy is operationalised, and to address the problematic tendency with existing studies of devolved health policy in the UK to rely solely on the self-reports of national-level policy actors (Smith *et al*., 2009; Stewart, 2013). Fuller details of our methods are provided in the online supplementary materials for this article. First, we analysed written guidance and regulations around major service change. Between May 2016 and February 2017, we conducted semi-structured interviews at the national level with 26 key individuals working on or around policy for public involvement in major service changes. Our interviewees (presented in Table 1) include civil servants in all four administrations; representatives of the intermediary bodies which ‘champion’ public involvement within each system; and relevant staff from other agencies with oversight of public involvement, major service change or both. To add context to our interviewees' perspectives, we sought to triangulate perspectives from interviews with documentary sources wherever possible.

**Table 1.** Interviews conducted

Professional location of interviewees	Number of interviewees	Total
Scotland		9
Scottish Government officials	1	
Senior staff at NHS agencies	3	
Experts with experience of policy advisory role	2	
Senior staff at two NHS Boards	2	
Public campaigner against service change	1	
England		11
Officials at Department of Health and NHS England	3	
Senior staff at intermediary body	1	
Experts with experience of policy advisory role	1	
Management consultants employed to advise on or deliver consultations	2	
Senior staff at PCT	1	
Senior staff at NHS Trust	1	
Staff at local intermediary body	1	
Public campaigner against service change	1	
Northern Ireland		13
Department of Health officials	2	
Senior staff at NHS agencies	3	
Experts with experience of policy advisory role	1	
Senior staff at intermediary body	1	
Senior staff at two NHS Trusts	4	
Public campaigners against service change	2	
Wales		12
Welsh Assembly Government officials	2	
Senior staff at NHS agencies	2	
Experts with experience of policy advisory role	2	
Senior staff at two NHS Trusts	2	
Public campaigners against service change	4	
**TOTALS**		**47**

On the basis of interviewee suggestions of change projects that they perceived as successful or unsuccessful, the research team selected two recent (in the last decade) case studies per country to explore further. Case study selection thus reflected interviewees' varied (and sometimes conflicting) understandings of what ‘good’ public involvement in change looked like (see Discussion for more on this). Where multiple potential case studies were available, we sought to include a variety of proposal outcome: whether the proposed change had been implemented, partially implemented or abandoned. Table 2 shows the key characteristics of our eight case studies, which have been anonymised at the request of several interviewees. The project was initially framed around hospital closures as a critical case – or as Timmins (2007) describes it, a ‘great taboo’ in the UK health policy – for involving the public in controversial decisions. As national fieldwork progressed, it became evident that (in part due to public and political opposition) full closures of hospitals are rare in some systems (particularly Northern Ireland), but also that other types of change were similarly contentious. Accordingly we broadened our scope, and our case studies include a mixture of major reconfigurations (e.g. closing multiple hospitals in Scotland A) and smaller change projects which nonetheless generated public opposition (e.g. closing a Minor Injuries Unit in Northern Ireland B). Our key interest was not in clinical definitions of the magnitude of change, but changes which are contentious with the public. In each case study, we sought interviewees to explore public involvement processes in practice, supplementing suggestions from previous interviews with Internet searches. The 18 case study interviewees include perspectives from key NHS staff responsible for involvement locally, but also from public interviewees. This is an effort not to represent the views of whole populations or understand the complex details of local politics, but to broaden the range of views we heard in order to better characterise policy. Campaigners against change often had quite different views from staff on the same processes. We used media searches and official documents to contextualise our understanding of the case studies.

**Table 2.** Case study details

Name of case	Population served by affected services	Area type	Change proposed	Change process outcome
Wales A	50–100,000	Rural	Removal of services and Accident & Emergency	Change abandoned
Wales B	Under 50,000	Rural	Removal of minor injuries and services including inpatient beds	Change partially implemented
Northern Ireland A	50–100,000	Urban	Closure of minor injuries unit	Change implemented
Northern Ireland B	Under 50,000	Semi-urban	Closure of specialist unit and Accident & Emergency	Change abandoned
England A	250,000–500,000	Urban	Closure of Accident & Emergency	Change abandoned
England B	500,000–1 million	Predominantly rural	Downgrade multiple Accident & Emergency to minor injuries units. Build new hospital	Change implemented
Scotland A	1 million +	Urban	Closure of multiple acute hospitals. Build new hospital	Change implemented
Scotland B	Under 50,000	Rural	Removal of inpatient beds and reduce Accident & Emergency to minor injuries service	Change ongoing with continuing contention

We analysed transcripts or written notes of interviews in NVivo software using an approach based on the Framework method (Ritchie and Lewis, 2003). We identified a list of key themes based on discussion among all four authors. These were: actors, rationales for involvement, stage of process, definitions of success and techniques. Authors 1 and 3 separately coded the same three transcripts to pilot the themes. Following further discussion, we amended and supplemented our themes (e.g. we added ‘underlying relationship’ as a ‘pre’ stage of the involvement process) and coded the rest of the data. We undertook respondent validation (see online supplementary materials) focused on checking for comprehensiveness and error reduction (Mays and Pope, 2000).

Ethical approval for the study was formally granted by Stewart's institutional ethics board. Given the sensitivity of some of our interviewees around changes which had attracted significant political and media attention, where quoting from interviews we have grouped interviewees into generic categories of professional location to avoid identifying individuals, specifying the location (England, Northern Ireland, Scotland or Wales) and allocating identifying numbers to individuals.

## 3. Findings: four systems' policy choices for public involvement in major change

In all four systems, major service changes, including hospital closures, were high on the agenda. Financial pressure was understood as simultaneously intensifying the need for change and making it harder to achieve:

“Nobody's pretending that we're swimming in cash and typically service change requires some resource. So I think the ambition and the reality of what is pragmatic is probably… there's probably quite a gulf there” (official 2, England).

Interviewees in all four systems referred to a ‘backlog’ of service change that they felt was necessary, but difficult to accomplish politically: many of the changes we heard about had histories predating current policy initiatives.

A policy requirement for NHS organisations to involve the public in proposed service changes was also consistent across all four systems. As identified in recent research on public involvement in major service change in Ireland (Foley *et al*., 2017), understandings of how and when to engage varied in practice. However, in our study, we were additionally struck by the different definitions of success operating within different change projects. Policy interviewees were not simply ‘pro’ or ‘anti’ (or ‘doing’, or ‘not doing’) public involvement in change, but had fundamentally different understandings of the role of public involvement work within change projects. There were also significant differences in how intensely Central Government scrutinised local change processes. Table 3 shows the formal process for involving the public in service change across the four systems. Some of these elements are statutory, and others are taken from written guidance. We have simplified certain elements to enable easy comparison for the reader (e.g. the English process mandates two phases of ‘assurance’ at distinct moments) and added details of clarification where they seem significant (such as the apparent hiatus of both the Welsh National Clinical Forum and the Scottish Independent Scrutiny Panels (ISPs)). In this section, we outline the four systems' approaches to encourage NHS organisations to involve the public in service change.

**Table 3.** Policy comparison

	ENGLAND	NORTHERN IRELAND	SCOTLAND	WALES
Who proposes major service change?	Clinical Commissioning Group	Trusts	Health Board	Local Health Board
How do national guidelines define quality engagement in change processes?	“Effective involvement means being open and transparent about proposals enabling local stakeholders to have the opportunity to influence change. Sometimes the most logical and well planned changes are not achievable due to inability to effectively involve the local population.” (NHS England, 2013, p. 14)	No guidance specific to service change but in general policy document: “PPI should be part of everyday working practice, underpinning communications and decisions regarding care or treatment. It should be an integral part of service planning, commissioning and delivery. It means discussing with those who use our services and the public: their ideas, your plans; their experiences, your experiences; why services need to change; what people want from services; how to make the best use of resources; and how to improve the quality and safety of services.” (Department of Health, Social Services and Public Safety, 2007)	“Public consultation about a service change should grow naturally out of a Board's everyday communication and dialogue with the people it serves. This guidance should support staff in their efforts to engage the public, and offer potentially affected people and communities a real opportunity to influence the Board's decision-making about the design and delivery of services through their involvement in: developing and appraising possible options to decide which should be the subject of a public consultation; and the public consultation on the preferred option(s).” (Chief Executive NHS Scotland, 2010)	“a further rebalancing between continuous engagement and formal consultation, with an even stronger emphasis on the former. The new NHS bodies and reformed Community Health Councils (CHCs) must work together to develop methods of continuous engagement which promote and deliver service transformation for their populations. It is not necessary to consult formally on every change that is required. Some changes can be taken forward as a result of effective engagement and widespread agreement.” (NHS Wales, 2011)
Who ‘assures’ quality public engagement has taken place for a specific change?	NHS England	No single body: Public Health Agency has oversight and Patient Client Council has a challenge function	Scottish Health Council (a Government agency)	Local Community Health Council
Who takes the final decision on change?	Clinical Commissioning Group	Trust	Board for most change. All major changes approved by Scottish Government	Trust (but cannot implement until CHC agrees)
Who can refer or call-in the decision for review?	Local Authority	Northern Ireland Executive	Scottish Health Council advises if a change is ‘major’: all major change is referred to Scottish Government	Community Health Council refers to Welsh Assembly Government
What are arrangements for independent scrutiny of decisions?	Independent Reconfiguration Panel: standing group with Department of Health secretariat	Equality Commission for Northern Ireland	Independent Scrutiny Panel can be convened by Scottish Government (none since 2009)	National Clinical Forum (last met in 2012)

In **England**, every civil servant we interviewed emphasised that if public involvement in major service change was going well, central Government would be unlikely to hear about it:

“If your starting point here is who's responsible for NHS service change … it doesn't take place in this building [the Department of Health] anymore. Government, central government is not responsible for service change in the NHS” (official 1, England).

The reference to ‘anymore’ here signals the reshaping of the Department of Health's role within English health care governance since 2012 (Exworthy and Mannion, 2016). Officials in England had minimal intelligence about reconfigurations before the point of things ‘going wrong’. This was partly because of the far larger population and complexity of organisational landscape, but it is important not to overplay the importance of England's sheer size here: politicians in the governments of all parties over decades have previously been closely involved in very local NHS matters.

Interviewees in England described seeking to move away from highly prescriptive guidance for public involvement. This was ostensibly to allow for tailored local solutions:

“I could send some guidance on the type of principles that [organisations] should apply, but actually the decision has to be made locally about what's best for the local population” (official 3, England).

However, it was also acknowledged to be an attempt to “keep politics out of it” (official 2, England). This seemed to refer specifically to keeping Ministers out of it, given that in both our English case studies the engagement of local MPs was critical to the respective outcomes.

In identifying solutions to their local problems, local actors were subject to informal oversight from NHS England

“They will look at their engagement plans and say whether they are involving the public in a way [NHS England] feel they should, but actually it's entirely their decision and they then take that forward themselves” (official 3, England).

That this review was only advisory was reinforced in written guidance “NHS England's assurance of service change proposals… offers advice and does not change accountabilities for decision making” (NHS England, 2013, p. 14). In the England B case, repeatedly described to us as a successful change project, it was noticeable that interviewees attributed their approach only to local expertise and commitment:

“It's good practice. There's a requirement… or there was a requirement on PCTs to engage in the planning and development of new services… but we began discussions around the engagement that *we* would like to see the trust do” (manager 2, England).

This had led to a communications and engagement plan heavily geared towards ‘selling’ the change proposals to the wider population:

“we had road shows, we have a rolling programme of engagement that we still do, so we take a big van, brand it up and we'll park ourselves in the town and… just deliberately engage and talk to people” (manager 1, England).

Commissioners in England have the option of seeking advice from Healthwatch, the intermediary body set up as a ‘consumer champion’. The intermediary role currently occupied by Healthwatch in England has been subject to greater change than in any of the other three systems (Baggott, 2005; Carter and Martin, 2016) and Healthwatch England, established in 2012, had its role and budget reduced in 2015 to one of coordinating and supporting local Healthwatch:

“a lot of that… was a bit top down and what [Healthwatch is] trying to do now is be very bottom up” (official 4, England).

Local Healthwatch branches must win contracts from local authorities and as Carter and Martin (2016) note they are easily overlooked: interviewees acknowledged that a key challenge for local Healthwatch branches was simply “being able to participate in the development of those plans and just being recognised” (official 4, England). This was also related to the use, much more widespread in England than in the other systems, of management consultancies to deliver consultations. The work these consultancies did was often well-regarded by staff:

“there's something about being able to say ‘look, this is an independent piece of research, it's not something that we've just done’. Plus, you know, you can guide people into answering the questions that you really want to answer and I think when you have an independent piece of research I think it just helps keep your barometer right” (manager 1, England).

However, in the England A case, the knowledge that profit was being made from what was seen as a box-ticking exercise had infuriated one campaigner interviewee:

“{Events] are very stage managed and private companies are paid huge amounts of money to run them” (member of public 1, England).

The Independent Reconfiguration Panel (IRP) was set up in 2003 as a standing body to advise the Minister on proposals for service change. Change proposals can be formally referred to the panel, but it can also be asked for advice and guidance. The panel comprises 15 members (managerial, clinical and lay), and a secretariat in the Department of Health. A 2015 review of the IRP's role considered the formal, standing organisational form as best-suited to providing ‘long-term, specialist, impartial and consistent advice’ (McMordie, 2015). As well as conducting partial or full reviews of proposals for service change, the IRP offers informal advice to NHS organisations and members of the public. While the IRP publishes regular ‘Learning from Reviews’ documents on how to conduct consultations well, it is primarily a ministerial advisor, not a public-facing organisation: it has no stand-alone website or social media presence. Interviewees praised the independence of the panel, but some expressed frustration that its significant learning was not having more impact on improving practice:

“The same things come up again and again [in processes referred to the IRP]” (expert 1, England).

**Northern Ireland**'s overall approach is legalistic when compared with those in the other systems, with a strong focus on organisations producing documents called ‘consultation schemes’ which set out the terms of consultation. Designed as a safeguard, these documents were frequently referred to in interviews as limiting potential for informal engagement:

“it's set in stone, we have to have a consultation scheme. Now, if say for example there was a service change and I thought I'll have a focus group… we're likely to be challenged… If it's controversial and somebody doesn't like our decision, which quite often happens, we would be challenged on the process and they would say ‘I had a legitimate expectation that I would be involved in that consultation process’ and a legitimate expectation of involvement is very much what is resulting in judicial reviews being successful” (manager 2, Northern Ireland).

Beyond statutory requirements, and unlike the other health systems, Northern Ireland does not have a single document with guidance for Trusts on public involvement in major service change specifically. More general policy guidance is, in its tone and language, not particularly accessible to a wider audience beyond key Trust decision-makers: an update around announcing emergency service changes (e.g. on safety grounds) is focused on legalities not on persuasively communicating information to a public audience (Department of Health, Social Services and Public Safety, 2012).

Staff described consultation fatigue due to the requirement to conduct formal consultations on even very small changes. In Northern Ireland B, staff argued that frequent consultation meant that

“when you actually need people to engage in something that you know will impact on the public and you need to have them on board with you, it's very difficult to get interest now because people are sick to the back teeth of consultations” (manager 3, Northern Ireland).

A previously abandoned consultation process in the same Trust years earlier had coloured campaigners' views of the process we studied:

“You know from experience that you're not going to be listened to, genuinely listened to. So, you know, it's soul destroying ‘cause you know you're just going through the motions. And it's a silly wee game that the Department of Health or the trust has asked you to play, when you know that the decision has more than likely been made anyway” (NI public 1).

We heard this weariness and cynicism from campaigners across all four systems, but it seemed to be particularly fuelled by the sheer frequency of consultations in Northern Ireland.

Northern Irish support and advice around public involvement is divided between the Public Health Agency (PHA) and the Patient Client Council (PCC): the former leading on support to Trusts in building involvement capacity and the latter offering ‘challenge’. A team within the PHA has the formal role “to champion the role of involvement from within the system” (official 6, Northern Ireland) but capacity to support Trust's involvement work was felt to be stretched, limiting their ability to get involved with specific local processes:

“we don't want to hold hands, and we're not in the position to hold hands at that level of detail” (official 6, Northern Ireland). This was borne out in Northern Ireland A case, where local staff stated “when we go to consult we're pretty much on our own as an organisation consulting unless, you know, sometimes we get a bit of support from the Board [of the Trust]. But it would be a bit surprising” (manager 2, Northern Ireland).

Other research has highlighted that public involvement capacity (in terms of specialist staff) is limited in Northern Irish Trusts, with public involvement duties often simply added on to existing roles (Duffy *et al*., 2017).

While the Patient and Client Council technically has a ‘challenge’ function regarding Trust service changes, it is more focused in practice on concerns from under-heard patient groups on province-wide issues. As with other intermediary bodies, demonstrating the independence of the PCC was seen as a challenge:

“There's room for co-operation absolutely and co-ordination [between Trusts and] the PCC, but if you're not careful… it could compromise the PCC. Number one they're funded by government and number two then if they started to conduct our involvement exercises you could have a significant question of their independence” (official 6, Northern Ireland).

When opposition to a proposed change escalated in one of our local case studies, the Trust requested that the regional agencies convene a ‘summit’ to find a solution, and the PCC was not one of the attendees. Northern Irish health policy attention has, over the last decade, focused on commissioning a series of international experts to recommend province-wide service reconfiguration, but implementation has been extremely patchy. In Northern Ireland B, one staff member predicted

“when [the Bengoa report] comes out, I mean, we know what it will say generally, well we don't *know*, but we know a sense of what it will say. But I know what it *won*'*t* say and it won't be really specific in particular areas and that'll be left for us to pick up those traits locally and to engage on how we change” (manager 3, Northern Ireland).

A key obstacle to moving ahead with reconfigurations has been the relative weakness or even absence of decisive political oversight of the Northern Irish NHS. Local staff described entrenched political nervousness about making change:

“because it's seen as contentious we have to get permission from [the Department of Health] to consult on the change in our organisation, because the Minister doesn't… indeed any Minister wouldn't like to be embarrassed by somebody breaking cover – and I've heard this term used – breaking cover on proposals” (manager 2, Northern Ireland).

Coupled with the consultation fatigue and stretched resources identified above, the Northern Irish NHS seems a very difficult place to change services.

**Scotland** is a significant outlier in terms of centre-local dynamics of service change with a highly prescriptive and explicitly interventionist approach from central Government. The 2010 service change process defines ‘major’ service change, and every ‘major change’ requires Ministerial approval, without any formal process of referral (Chief Executive NHS Scotland, 2010). Thus the Scottish Government has oversight of all significant changes in process, regardless of whether anything has ‘gone wrong’ in the consultation. Many interviewees in Scotland argued that this commitment to public involvement within policy had slowed the pace of service change: written guidance states that the views of potentially affected communities should be “treated with the same priority (unless in exceptional circumstances e.g. patient safety) as clinical standards and financial performance” (Chief Executive NHS Scotland, 2010, p. 4). Only one major service change proposal was rejected between 2010 and 2016; it seems that Boards, anticipating Ministerial scrutiny, have tended to avoid changes that might incur it.

Closely related to the extent of central government involvement in change processes was the prescriptiveness of guidance issued to local organisations. Scotland has the most detailed guidance specifically on public involvement in major service change which sets out a three-stage process including public representation in a formal process of option appraisal (Chief Executive NHS Scotland, 2010; Scottish Health Council, 2010*a*, 2010*b*). This was perceived positively at the national level as having ‘worked’:

“The processes in Scotland, give the boards almost a level of protection… if they go through that process and do it right, they're getting to a point at which their consultation process stands up to that challenge” (official 1, Scotland).

There were frustrations locally from some managers. In Scotland case A, one manager felt the process lacked ambition:

“it was set up as a mechanism to ensure that the technicalities of any government guidance were adhered to… I don't think they have been able to nor are they in a position to take that bigger view” (manager 1, Scotland).

In case A, though, there was a sense that following the process would make Ministerial approval likely:

“incredibly nitpicky… it was absolutely exhausting… [But] you're much more certain to be able to make the change if you've gone through the process, you've got a fighting chance” (NHS manager 2, Scotland).

Perhaps surprisingly, the exhaustive nature of the prescribed change process was not necessarily popular with public interviewees in Scotland B:

“this steering group has gone on all these years ever since talking about this and it's really getting nowhere” (member of public 1, Scotland).

However, because there is little clarity about the Minister's decision-making process, following the prescribed process and achieving a positive written report from the Scottish Health Council was seen as Boards' best chance of achieving change. Assuring the ‘quality’ of public involvement in change proposals has not been without its tensions. Concerns in the late 2000s led to an independent review of the Scottish Health Council and the centralisation of service change expertise into the national office, where previously it had been a function of local offices:

“It wasn't great with that assessment role sitting with an improvement role, you know, staff going to the boards 1 day saying ‘we're here to support you’ the next they're going and saying ‘now we're going to assess what you've done’” (official 4, Scotland).

Removing the service change role from local offices was felt by national interviewees to have allowed local officers to improve their relationships with Boards, while the national Service Change Team became a more specialised resource for difficult changes. Scottish policy also allows for ISPs Independent Scrutiny Panels (ISPs) to be convened at the request of the Minister, with an ad-hoc secretariat set up within the Scottish Health Council. Three panels (comprising 3–4 experts and stakeholders) took place between 2008 and 2010, but none has been convened since (and neither of our case studies was subject to reviews). ISPs were described by several interviewees as expensive and time-consuming, but ‘an effective backstop’ (official 3, Scotland) which had sent a message to Boards to take public involvement seriously.

**Wales**'s current approach is most distinctive in its aspirations, with guidance repeatedly calling for ‘a further rebalancing between continuous engagement and formal consultation, with an even stronger emphasis on the former’ (NHS Wales, 2011). This desire to embed a collaborative relationship with the public into health care decision-making led officials to avoid highly prescriptive guidance in favour of a focus on ‘culture change’:

“We're taking a view that perhaps detailed written guidance ‘do this, do that’ is too paternalistic and mitigates against actually successfully changing your culture…” (official 1, Wales).

This policy shift followed decades of contentious decisions, with both of our Welsh case studies demonstrating a legacy of multiple reconfigurations abandoned due to public opposition (and appeals to Ministers).

However, the Welsh Assembly Government's role was more hands-on than in England, and interviewees in Wales explained that civil servants tended to have a ‘light touch oversight’ of change processes. While the risk of judicial review meant that Ministers were keen to avoid close engagement with decision-making, officials played a mediating role, in some cases ‘help[ing] to facilitate and broker a discussion and agreement’ (official 1, Wales) between local actors to avoid the need for a formal referral to the Minister. Indeed there were several examples, including Wales case A, of innovative attempts to bring managers, clinicians and trenchant campaigners together to make decisions brokered by Government. This collaborative approach is time-consuming to develop and has proved difficult to implement:

“engagement is hard, it takes a lot of work, it takes a lot of evening meetings, it takes a lot of one to ones with the stakeholders” (manager 2, Wales).

The slow pace of change was frustrating for many health system insiders. Referring again to the long-term collaboration with campaigners in case study A, one interviewee stated

“It had excellent engagement, consultation… I think the only problem I've got with it is the pace” (official 1, Wales).

In Wales the local, volunteer-led Community Health Council (CHC) must approve any major change proposals and is the only body that can refer them to the Minister. CHCs have been repeatedly criticised in reviews for lacking visibility with the public and for variable quality (Lloyd, 2014; Longley *et al*., 2014); in 2017, the Welsh Assembly Government consulted on abolishing them. One key critique related to their role in service change proposals, when some local CHCs had become involved in conducting their own consultation activities:

“carrying out their own engagement with the public on the proposals that had been put and then process[ing] that intelligence and represent[ing] it to the health board in the way they thought appropriate” (Expert 1, Wales).

This was perceived as complicating the CHC's assurance role; one CHC was itself subject to a judicial review of its decision *not* to refer a service change to the Minister (Smith, 2014). The local CHC was not highly-regarded in Wales case B with one campaigner describing the local branch as ‘well in with the board members’ (member of public 2, Wales). In Wales case A, though, one manager praised the local CHCs work eliciting patient views:

“they do spend an awful lot of time talking to the public and they do get some really good feedback from service users that maybe other inspectorates don't get because they haven't got the time” (manager 2, Wales).

Despite collaborative aspirations, service change remained a contentious and politicised issue in Wales. A Welsh body called the National Clinical Forum was established in 2011 (with a broadly similar role to the IRP in England) but lacking lay representation. It did not manage to establish the reputational strength of the IRP, and its impartiality was almost immediately questioned when it was accused of rewriting a report under pressure from a Board (BBC News Online, 2012). Its profile has been so low since then that one well-informed interviewee was unsure whether it still existed; its web page was last updated in August 2012 and we were unable to contact members.

## 4. Discussion: carrots, sticks and sermons

This study illuminates the difficulty of straightforwardly evaluating policy and practice in the contentious area of public involvement within health care change (for more on the limits of evaluation see Marsh and McConnell, 2010; Jones, 2018). The perceived purposes of public involvement in major service change vary not merely between campaigners and decision-makers, but between different publics and at different levels of the health system. Success might also be assessed at different moments in the decision-making process: the absence of judicial review, in long-term goal achievement (a hospital closed), in eventual population health outcomes, or indeed in laying the ground work for a trusting relationship between organisation and population. Both because success is contested and because change projects continue to evolve beyond the point of ‘implementation’, in Table 2 we list only an ‘outcome’ for each case at the point of data collection.

The scope and depth of this study does not permit a declaration of ‘what works’ in this complex area of health policy: we examined only a small and purposively selected sample of case studies. Instead, in this policy-focused paper, we emphasise choices made at the health system level in different policy environments and between different actors. This recognises the value of exploring alternative framings of a policy where issues are intrinsically contested and where conflict is entrenched (Schön and Rein, 1994). When we asked policymakers to suggest successful case studies, their answers reflected the priorities of the system they worked within, rather than a coherent or stable account of public involvement ‘best practice’ (Contandriopoulos, 2004). In Wales multiple interviewees suggested Wales A which had, through collaborative practices, begun to repair the legacy of distrust from previous service changes, but which had to date delivered no substantive solution to how services could be sustainably managed. In England's climate of urgency, we were repeatedly pointed towards England B which at the time of data collection was held up nationally as a slick operation which had effected radical change (although where in the intervening years, implementation has stalled).

The emphasis on organisational behaviour currently prevalent in much change management and public involvement literature risks neglecting the ways in which health care organisations respond to the context of their health system, taking cues and selecting priorities on the basis of the ‘carrots, sticks and sermons’ (Bemelmans-Videc *et al*., 2003) of policy (Pettigrew *et al*., 1988; Powell, 2016). Our four-system study allows us to compare the political and policy context of health systems, including both specific policy tools selected by Governments, and how the architecture of the wider health system constrained and promoted particular forms of practice around public involvement. Organisations were learning and developing practices of public involvement within these system-specific parameters.

In terms of system factors, collaborative work and the creation of trusting relationships to enable dialogue seemed easier where organisations were stable over time. Fragmented health systems, particularly England, where multiple organisations operated across a population, seemed to distract attention from building the kind of ongoing collaborative relationships with publics that Welsh policy, in particular, prized. The organisational anxiety and turmoil of periods of rapid reform was not conducive to organisations working with the public towards service transformation. Carter and Martin's (2018) ethnographic study in England identifies how well-intentioned involvement activities were circumscribed by system actors' urgent need to make significant cost savings in response to national policy. Top-down reorganisations of the English NHS have thrown up both practical issues for public involvement (public confusion about who makes decisions) and motivational ones (organisations understand that their survival is dependent on central Government, not their local populations). The differing level of functional autonomy health care organisations have from central government in each system is also significant. The proximity of politics to day-to-day NHS decision-making in the devolved systems is undeniably greater. However, even in the sometimes ‘hierarchist’ (Schang and Morton, 2017) devolved health systems national actors cannot *ensure* that organisations will take a particular approach to involving the public in contentious change.

In none of the health systems did we see the overt use of policy ‘carrots’ – subsidies – to encourage organisations to involve the public in service change. Decisions whether to properly fund agencies which are tasked with developing better public involvement could be understood as an incentive, and in particular where this work had been under-resourced (as we heard in Northern Ireland and England) the lack of investment suggested that involving the public in service change was a relatively low priority.

By contrast the use of policy ‘sticks’ to mandate health care organisations to involve the public was present in all systems, and most visible in Scotland since the late 2000s. As described above, opinions on the efficacy of this approach were divided in Scotland, but reversing unpopular decisions, implementing prescriptive guidance, imposing independent scrutiny and ensuring Government oversight of any major change had underlined governmental commitment to public involvement in the NHS. Northern Ireland's highly legalistic approach can also be read as stick-focused, or at least focused on mandating particular action under fear of judicial review. In both these systems, reliance on these hierarchical sticks risked drawing the attention of organisations back towards managing their relationship with Edinburgh or Belfast, not developing relationships with their local population. Likewise, campaigners had less incentive to engage with local consultation processes and more to ‘venue shop’ (Ansell and Gash, 2008) with elected politicians, knowing that the eventual decision would not be made locally.

‘Sermons’ or information tools include the skills development and supportive aspects of policy around public involvement in local service changes. This includes ongoing advice and support between local organisations and either intermediary bodies or officials. A ‘sermons’ approach implies that organisations need to be persuaded to enact particular forms of public involvement, and in this policy area acknowledges the importance of organisational actors not merely ‘going through the motions’ of involvement. In this study, the deficit was sometimes interpreted instead not as a lack of willing, but a lack of skills. While we heard repeatedly that public involvement in major service change is ‘not rocket science’ (official 3, Wales), interviewees did emphasise the value of support in planning this activity, especially since the experience of conducting public involvement on unpopular service changes can be bruising and off-putting. In practice, while support is currently available across all four systems, the degree to which it was adequately resourced, and the form it took, varied significantly. The locally-led approach of the CHCs in Wales meant that the support was variable between areas. In Scotland, locating this expertise within the national office of the Scottish Health Council allowed for consolidation in an expert team (rather than it being a minor part of a wider public involvement role). In England, the advisory function of the IRP, while well-regarded by our interviewees, was complicated by the fact that organisations rarely consulted their guidance before reconfigurations were ‘going wrong’, and it was notable that at the local level capacity-building support was often being ‘bought in’ from management consultancies. In Northern Ireland, the support and advice available was complicated by being split between the Patient Client Council and the Public Health Agency, and was perceived to be under-resourced in both organisations.

The relative balance [or ‘packaging’ (Bemelmans-Videc *et al*., 2003)] of carrots, sticks and sermons in each system is a complex result of history, political expediency and macro-level factors such as the budgetary constraint which all four health systems had experienced, but combinations of policy tools were significant. For example, the ‘sermons’ offered by the Scottish Health Council were more influential because the ‘sticks’ discussed above encouraged Boards to seek their advice early and take it seriously. In England, the ‘sermons’ emanating from NHS England, Healthwatch and the IRP were much less influential because managers were under massive pressure from elsewhere in the system to make change quickly. The ‘sticks’ of Northern Irish regulation, without fully-resourced ‘sermons’, made for less thorough consultations, and for significant ‘consultation fatigue’. In Wales, aspirations towards collaborative working were somewhat stymied by a relative lack of both sticks and sermons, and the legacy of adversarial ‘sticks’ which encouraged local actors to look to the Minister for resolution of conflict.

Across these packages of policy tools in each system, there was variation in the extent to which policy was prescriptive in telling NHS organisations not merely that they *should* involve the public, but *how* they should do so. In Scotland, the Scottish Health Council had taken an already prescriptive and detailed policy (the document ‘CEL 4’ (Chief Executive NHS Scotland (2010))) and developed a set of matrices and guidance which acted to institutionalise a particular vision of what good public involvement entailed (prolonged, with multiple stages of involvement including formal options appraisal). The provision of a tailored report on each service change to the Cabinet Secretary further encouraged Boards to toe this line. In no other system in our study was a single team of this size developing an overview of the policy area, let alone being accorded a significant role in the process. Organisations in the other systems were either receiving more general advice from a smaller and more stretched central agency, or were being encouraged to find their own approach, drawing on advice from a range of organisations inside and outside the public sector. This space could either create opportunities for local advocates of involvement to experiment and innovate, or allow public involvement to slip back down the pressured list of NHS priorities.

## 5. Conclusion

Cross-system studies of public involvement in health are challenging and rare (Slutsky *et al*., 2016), but in the UK, our research suggests that Governments, while frequently espousing broadly collaborative visions of involvement, both struggle to have organisations enact them at the local level, and often make wider decisions which make those visions more difficult to realise. Where the organisational landscape is chaotic and fast-changing, as in England, rapid ‘transformation’ was valued. In Scotland and Wales, where there has been a greater degree of organisational stability, we heard more about longer-term projects of collaborative change, although this was by no means consistent and brought its own frustrations. In the Northern Irish landscape, Trusts rarely made proposals for significant changes to hospital services, and tended to rely on traditional, legalistic approaches to consulting the public where changes seemed necessary. In all three devolved settings, policy and intense political oversight discouraged managers from making daring changes relative to England, where both scale and Ministerial detachment made initiating change, if not convincing local publics, look easier.

Prescriptions for the future shape of health systems from health policy elites express urgency and great potential for public benefit from transformed health systems (Crisp *et al*., 2016). However, realising the potential gains from transforming health systems is, at least in democratic systems, properly dependent on wider public support for the changes that are required. As in other examples where expert-led developments encounter widespread public opposition, the particular character of attempts at public engagement are crucial in finding acceptable ways forward (Aitken, 2010; Chilvers and Kearnes, 2015; Stewart and Aitken, 2015; Allen *et al*., 2017). It is frequently argued that organisations simply need to conduct ‘meaningful’ engagement in order to change services effectively (Dalton *et al*., 2016; Foley *et al*., 2017). However, our study suggests that, even at the national level, there is little consensus on what constitutes success in this area, and that even where a clear policy goal is identified (e.g. continuous engagement in Wales), health policy is not well-equipped to create it at the local level.

## Supplementary material

For supplementary material accompanying this paper visit https://doi.org/10.1017/S1744133119000148. click here to view supplementary material
