# Supplementary material for: Transforming health care: the policy and politics of service reconfiguration in the UK's four health systems
Source: Health Econ Policy Law. 2019 Apr 12;15(3):289–307. doi: 10.1017/S1744133119000148 (PMC7525102; doi:10.1017/S1744133119000148)
Supplement: Supplementary file 1 [file S1744133119000148sup001.docx]

**Methods supplementary information for ‘Transforming healthcare’ article**

Policy documents analysed are listed in table a. Our tasks including understanding key differences in terminology between the four systems. These documents were identified by web searching and later supplemented with suggestions from interviewees.

These searches were supplemented with snowballing and advice from our research funder to identify key individuals and organisations to approach for interview in each system. Details of interviewees are given in table 1 (main article). The only key gaps in our interviewing are the national Board of Community Health Councils or the National Clinical Forum in Wales (both of which we made repeated efforts to contact). Of 26 national interviewees, 9 were interviewed by author 1 and 17 by author 2. 18 interviewees were interviewed in person and eight by telephone. At the request of the interviewees, 4 were interviewed in pairs with a colleague. Interviews were audio-recorded except for two cases where the device failed and one where the interviewee did not agree to recording: detailed notes from these interviews were taken. Questions covered the interviewee’s role in public involvement and major service change, key developments in policy for involving the public in service change, perceived strengths and weaknesses of the current system, and finally, examples where involving the public in major service change had gone ‘well’ or ‘badly’.

We conducted interviews across all case studies; identifying staff who had played key roles in the consultation process and members of the public who had been vocally opposed to the proposals. However case study recruitment was more challenging than at the national level. Staff in cases perceived as having gone badly were often reluctant to take part, and in several cases we struggled to contact members of the public active in the process. For case study interviews, 7 people were interviewed by author 1 and 11 by author 3. 3 interviews were conducted by telephone, and the remaining 15 in person. One interview was a group interview with 3 public campaigners. Interview questions covered experiences of the change process, perceptions of what had gone well or badly, and interviewees’ awareness (if any) of the role of national guidance within that.

Following analysis we shared a briefing of preliminary findings with key individuals from the four systems in a roundtable event in September 2016. We then sent a more developed draft briefing of findings and conclusions to all interviewees in December 2016, and presented the findings at a public seminar in January 2017., as well as allowing us to test our interpretations. Feedback highlighted an organisational role we had misunderstood in Northern Ireland, and an alternative interpretation of some of our findings in England. Author 1 conducted a further three interviews to explore these issues further in February 2017 (these interviews are included in the discussion of national interviews above).

Table A: Key policy documents

| England | Northern Ireland | Scotland | Wales |
| --- | --- | --- | --- |
| NHS England/ Operations and Delivery. (2015) *Planning, assuring and delivering service change for patients*.  NHS England/ Operations and Delivery. Available at: <https://www.england.nhs.uk/wp-content/uploads/2015/10/plan-ass-deliv-serv-chge.pdf> | Belfast HSC Trust. (2010)*Equality scheme: Drawn up in accordance with section 75 and schedule 9 of the Northern Ireland Act 1998*. Belfast HSC Trust. Available at: <http://www.belfasttrust.hscni.net/pdf/EQIA_OPHTHALMOLOGY.pdf> | Woods, K. J. (2010, October 2). Informing, engaging and consulting people in developing health and community care services. Chief Executive’s Letter. Retrieved from http://www.sehd.scot.nhs.uk/mels/CEL2010_04.pdf | Welsh Government, 2011. NHS Wales guidance on engagement and consultation. [pdf] Welsh Government. Available at: <http://www.wales.nhs.uk/sitesplus/documents/829/nhs%20wales%20guidance%20on%20engagement%20and%20consultation.pdf> |
| Independent Reconfiguration Panel (IRP). (2015*) Independent Reconfiguration Panel: The review process.* Independent Reconfiguration Panel. Available at:  <https://webcache.googleusercontent.com/search?q=cache:PtAvWUS0jksJ:https://www.gov.uk/government/uploads/system/uploads/attachment_data/file/461570/000_The_review_process_17.09.15.doc+&cd=1&hl=en&ct=clnk&gl=uk> | Equality Commission for Northern Ireland. (2005)  *Section 75 of the Northern Ireland Act 1998: Practical guidance on equality impact assessment.* Equality for Northern Ireland. Available at: <http://www.equalityni.org/ECNI/media/ECNI/Publications/Employers%20and%20Service%20Providers/PracticalGuidanceonEQIA2005.pdf> | Scottish Health Council. (2010a). *Identifying Major Service Change*. Glasgow: Scottish Health Council. Retrieved from http://www.scottishhealthcouncil.org/about_us/what_we_do/service_change/identify_major_service_change.aspx#.WAyfVkb09qw | The Welsh NHS Confederation, 2015. *The 2016 challenge: A vision for NHS Wales*. Cardiff: The Welsh NHS Confederation. |
| Independent Reconfiguration Panel (IRP). (2010) Learning from reviews, third edition. Available at: https://www.gov.uk/government/publications/learning-from-reviews-third-edition | Department of Health, Social Services and Public Safety. (2007). Guidance on Strengthening Personal and Public Involvement In Health and Social Care. Retrieved from https://www.health-ni.gov.uk/sites/default/files/publications/dhssps/HSC%20%28SQSD%29%2029-07_2.pdf | Scottish Health Council. (2010b). *Involving Patients, Carers and the Public in Option Appraisal for Major Health Service Changes*. Glasgow: Scottish Health Council. Retrieved from http://www.scottishhealthcouncil.org/about_us/what_we_do/service_change/option_appraisal.aspx#.WAyfoEb09qw | Welsh Government. (2011). *Together for Health: a five year vision for the NHS in Wales*. Cardiff: Welsh Government. Retrieved from http://gov.wales/docs/dhss/publications/111101togetheren.pdf |
| NHS England. (2014). *Five Year Forward View*. Leeds: NHS England. Retrieved from https://www.england.nhs.uk/wp-content/uploads/2014/10/5yfv-web.pdf | HSC Public Health Agency. (2012). *Valuing people, valuing their participation: a strategy for Personal and Public Involvement for the Public Health Agency and Health and Social Care Board*. Belfast: Public Health Agency. Retrieved from http://www.publichealth.hscni.net/sites/default/files/PPI%20Strategy%20-%20March%202012_0.pdf | Scottish Health Council. (2015). *Service Change Flowchart*. Glasgow: Scottish Health Council. Retrieved from http://www.scottishhealthcouncil.org/about_us/what_we_do/service_change/service_change_flowchart.aspx#.WmDbNGefZqw | McAllister, F., & Blunt. (2013). The People’s NHS: Research to support the consultation on creating a ‘Compact’ with the people of Wales in relation to their health and health services (Government Social Research No. 24/2013). Cardiff: Welsh Government. Retrieved from http://gov.wales/statistics-and-research/peoples-nhs/?lang=en |
|  | Livingstone, J. (2012). *Guidance for HSC Organisations on Arrangements for Implementing effective personal and public involvement in the HSC* (Policy circular No. HSC (SQSD) 03/2012). Belfast: Department of Health, Social Services and Public Safety. Retrieved from https://www.health-ni.gov.uk/sites/default/files/publications/dhssps/HSC%20%28SQSD%29%2003-12_0.pdf | Scottish Government. (2016). *A National Clinical Strategy for Scotland* (Report). Edinburgh: Scottish Government. Retrieved from http://www.gov.scot/Publications/2016/02/8699 |  |
